# Multiplanar Stability After Syndesmotic Screw Fixation vs Deltoid Ligament Repair in a Cadaveric Weber B/SER4b Ankle Fracture Model With Fibular Plate Fixation

**DOI:** 10.1177/10711007261445948

**Published:** 2026-06-10

**Authors:** Esten K. Ø. Haanæs, Andreas F. Dalen, Martin G. Gregersen, Aleksander Skrede, Marius Molund

**Affiliations:** 1 Department of Orthopaedic Surgery, Østfold Hospital Trust, Grålum, Norway; 2 Department of Neuromedicine and Movement Science, Norwegian University of Science and Tehcnology, Trondheim, Norway; 3 Department of Orthopaedic Surgery, Helse Nord-Trøndelag Hospital Trust, Sykehuset Levanger; 4 Norwegian Armed Forces, Medical Division, Sessvollmoen, Norway; 5 Department of Research and Innovation, Møre and Romsdal Hospital Trust, Ålesund, Norway; 6 Department of Orthopaedic Surgery, Møre and Romsdal Hospital Trust, Ålesund, Norway; 7 Department of Physical Medicine and Rehabilitation, Østfold Hospital Trust, Grålum, Norway; 8 Institute of Health and Society, Faculty of Medicine, University of Oslo, Oslo, Norway; 9 Department of ICT and Natural Sciences, Norwegian University of Science and Technology, Ålesund, Norway

**Keywords:** ankle fracture, Weber B, biomechanics, stability assessment, trauma, deltoid ligament, syndesmotic injury

## Abstract

**Background:**

Weber B / Lauge-Hansen supination-external rotation 4b (SER4b) ankle fractures are unstable injuries characterized by complete deltoid ligament ruptures. Biomechanical studies suggest that fibular plate fixation alone is insufficient for SER4b fractures. Concurrent deltoid ligament repair seems necessary. This study explores how syndesmotic fixation, which is a simpler surgical approach, restores stability compared to deltoid repair or combined.

**Methods:**

Eight fresh frozen human cadaveric ankle specimens were tested sequentially by a robot in all 5 states: native, SER4b injury model with anatomic fibular plate fixation only, or reinforced with a syndesmotic screw and/or deltoid ligament repair. Stability was measured in lateral translation (talar shift), valgus, and internal and external rotation during a constant 45-N axial load. The primary outcome was talar shift on fluoroscopic images. The minimal clinically important difference (MCID) was predefined as 1.0 mm.

**Results:**

Combined plate and syndesmotic screw fixation restored lateral translation stability. The mean difference in talar shift was −0.2 mm compared with native (95% CI −0.8 to 0.5, *P* = .57). However, talar valgus tilt increased by 18.1° (95% CI 16.1-20.0, *P* < .001) and external rotation by 15.9° (95% CI 12.6-19.2, *P* < .001). For combined plate fixation and deltoid ligament repair vs native, the mean difference in talar shift was 0.3 mm (95% CI −0.4 to 1.0), *P* = .37), also below the pre-defined MCID. Mean valgus and external rotation deviated less from native but were still significantly different, the respective mean differences were 2.7° (95% CI 0.8-4.7, *P* = .006) and 5.2° (95% CI 1.9-8.5, *P* = .002).

**Conclusion:**

In this cadaveric SER4b injury model, both syndesmotic screw fixation and deltoid ligament repair restore lateral translational stability, when combined with fibular plate fixation. However, only deltoid ligament repair—addressing both the deep posterior and anterior deltoid components—substantially restored rotational and valgus stability, suggesting it provides more comprehensive joint stabilization than syndesmotic screw fixation alone.

**Clinical Relevance:**

Syndesmotic screw fixation restores lateral translational stability as well as deltoid ligament repair when both are combined with lateral plate fixation. Complete deltoid ligament repair is superior to the syndesmotic screw in restoring overall stability in cadaveric SER4b ankle fracture models. Given their potential clinical relevance, these findings may be considered in future clinical studies and choice of treatment.

## Graphical abstract

**Graphical Abstract:**
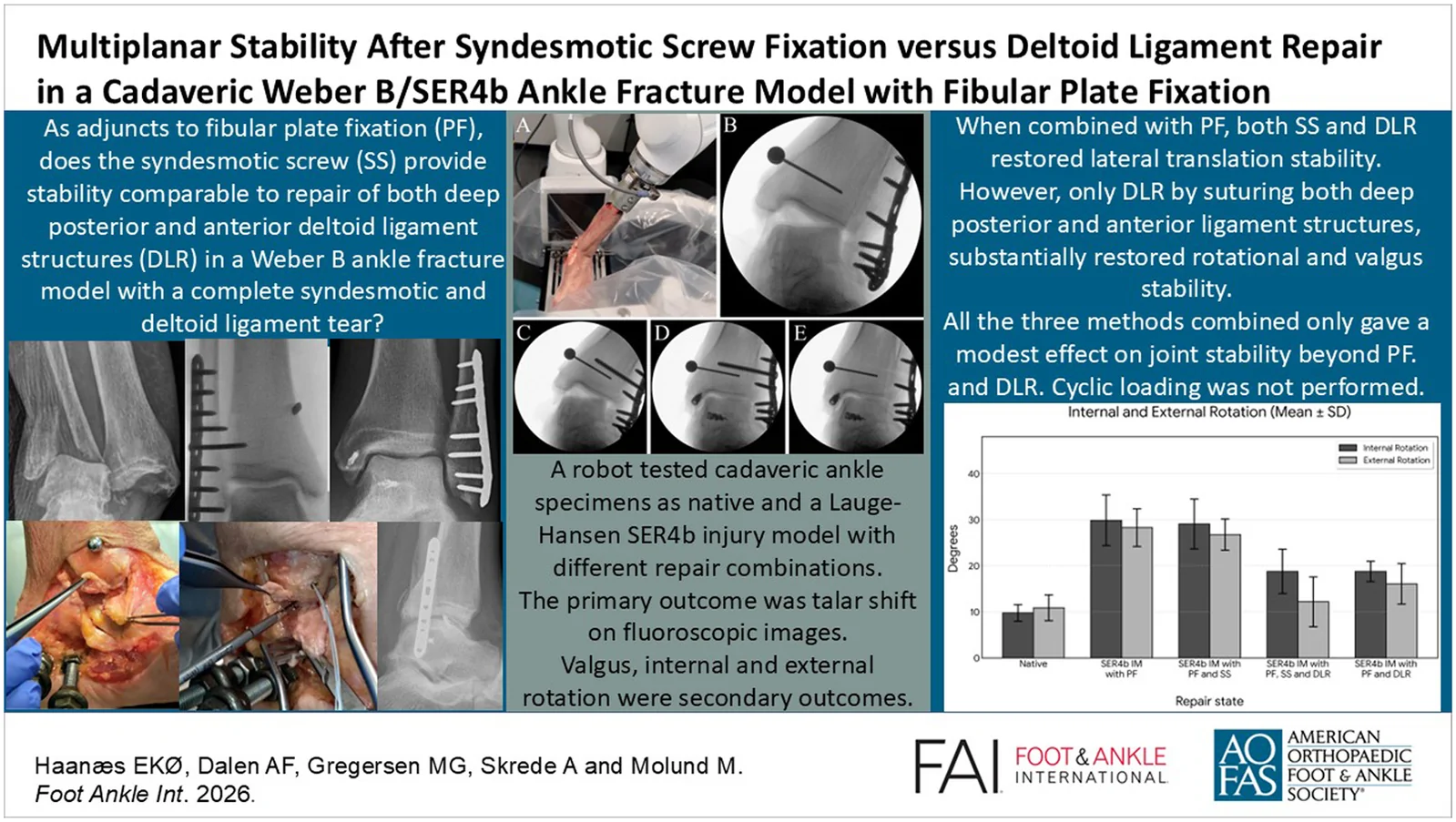
This is a visual representation of the abstract.

## Introduction

Weber B / Lauge-Hansen supination-external rotation (SER) ankle fractures can be classified by the degree of deltoid ligament injury as intact ligament (SER2), partial ligament rupture with an intact deep posterior tibiotalar ligament (SER4a), or complete ligament rupture (SER4b).^1-3^ Distinguishing between partial and complete deltoid ligament ruptures may define the threshold for surgical intervention. Traditionally, ankle fractures with partial deltoid ruptures (SER4a) have been managed operatively by plate fixation of the lateral malleolus and a syndesmotic stabilisation when considered beneficial.^4^ However, multiple clinical and biomechanical studies have demonstrated that SER4a fractures are often sufficiently stable for nonoperative treatment, with excellent reported outcomes.^2,5-7^

Most clinical studies on surgical treatment of unstable ankle fractures at the level of the joint (Weber B fractures) group SER4a and SER4b injuries together. Several reports on complete deltoid ligament injuries also involve the more proximal (Weber C) pronation-external rotation (PER) and pronation-abduction (PAB) injuries.^8-10^ Clinical data on carefully selected SER4b fractures are scarce, limiting our understanding of the specific treatment needs of SER4b fractures. Consequently, the optimal management strategy for SER4b remains uncertain. Three previous biomechanical studies show that fibular plate fixation alone did not fully restore ankle joint stability in an unstable Weber B (SER4b) injury model.^11-13^ Additional repair of components of the deltoid ligament was found to be necessary to restore stability.^11-13^ Only a few clinical studies from recent years state inclusion criteria appropriate to select SER4b injuries. Unfortunately, descriptions of the different injury patterns observed, their relative frequencies, and how they are repaired are poor or lacking.^8,10,14^ Deltoid ligament repair may be technically demanding and requires an additional medial incision. An alternative but technically simpler approach is to combine fibular plate fixation with trans-syndesmotic fixation (eg, syndesmotic screws or syndesmotic buttons).^4,15,16^ This raises important clinical questions:

1. Does a combination of syndesmotic screw and fibular plate fixation provide stability comparable to that achieved with combined fibular plate fixation and deltoid ligament repair?
2. When both a deltoid ligament repair and syndesmotic screw fixation are performed in addition to fibular plate fixation, does this combination offer additional stability?

The purpose of this biomechanical cadaveric study is to evaluate the immediate stabilizing effect of the 3 different repair combinations in a cadaveric SER4b injury model.

## Materials and Methods

### Test Samples

Eleven fresh frozen human cadaveric foot and ankle specimens (Science Care) were acquired for this study. Donors had given consent to donation for research purposes, and no history of foot or ankle injury, osteoarthritis, or surgical procedures had been reported. Investigations were performed at Ålesund Biomechanics Lab, Ålesund, Norway. Ethical approval for the study was given by the Regional Committee for Medical and Health Research Ethics (reference number 178067).

### Specimen Preparations

**Figure 1.**
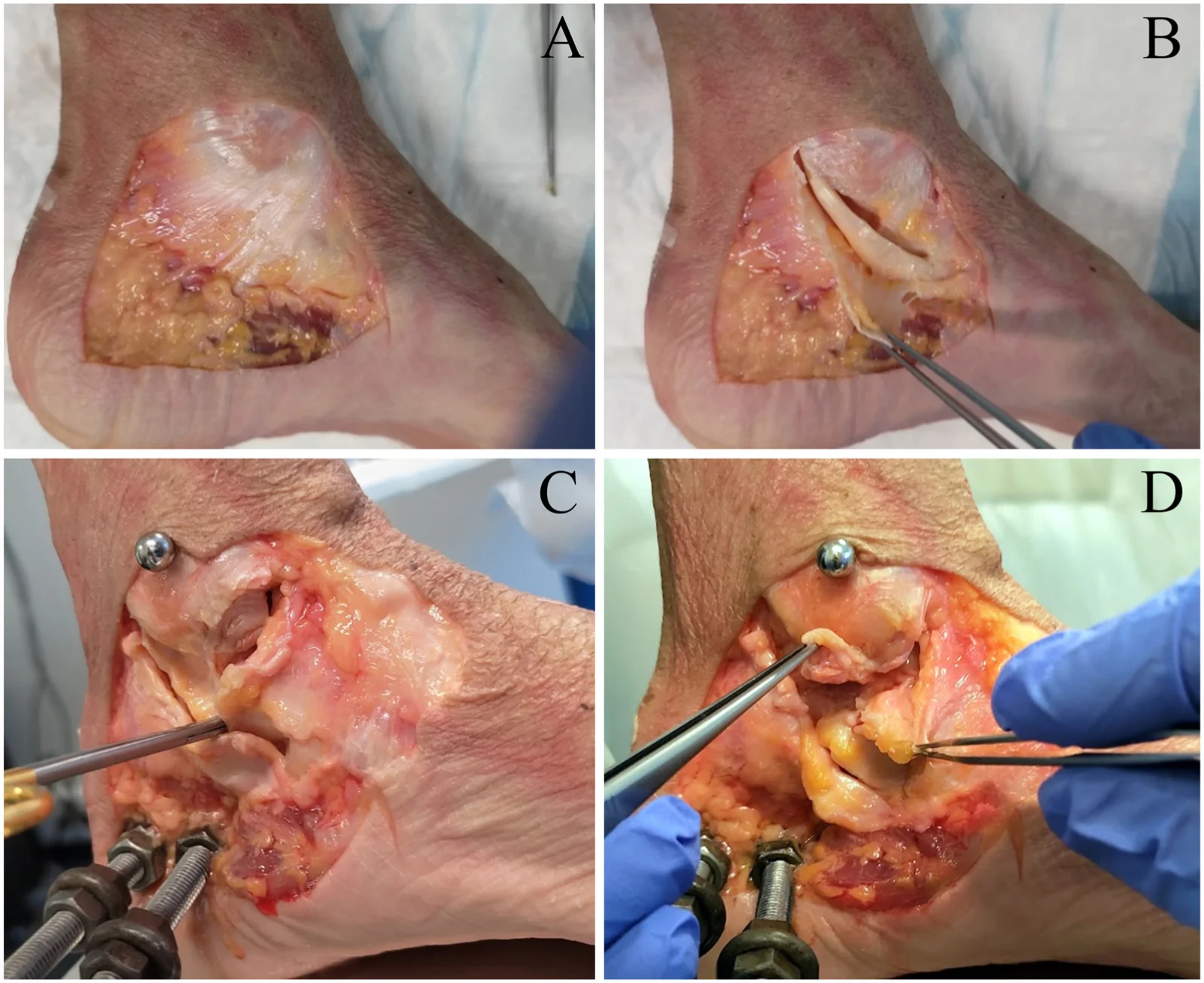
Dissection of the medial aspect of the ankle and creation of the deltoid ligament injury model. (A) The medial flexor retinaculum. (B) The retinaculum has been dissected off the posterior and distal part of the medial malleolus exposing the tibialis posterior tendon. (C) After removal of the tendon, anterior parts of the deltoid ligament have been dissected from the colliculus anterior of the medial malleolus. Scissors placed in the subtalar joint for orientation. (D) Posterior part of the deltoid ligament dissected from the medial side of the talus, a pragmatic simplification of the most common injury pattern as described by Jeong et al.^18^

The foot and ankle specimens were stored at −23°C and thawed for a minimum of 12 hours prior to preparation and testing. The proximal tibial end was cut approximately 20 to 22 cm from the distal tip of the medial malleolus, depending on the length of the specimen, and cast with resin (Rencast FC 52/53 Isocyanate, Polyol FC 53, Filler DT 082; Huntsman Corp) into a metal cylinder that fit the attachment on the distal robotic arm. The calcaneus was reinforced with resin through a lateral exposure. Two holes were predrilled for threaded rods (M8, stainless steel), further connected to the testing platform by eyebolts and nuts. On the medial side of the ankle, a window with skin and subcutaneous tissue was removed, as described in former studies.^6,11,17^ Portions of the medial flexor retinaculum and the tibialis posterior tendon were excised to ensure adequate exposure. The deltoid ligament was carefully exposed for subsequent transection (Figure 1). The consultant foot and ankle surgeon and the head of the biomechanic lab, who is a consultant general orthopaedic surgeon, performed all dissections, creation and repair of injuries.

### Test Setup

**Figure 2.**
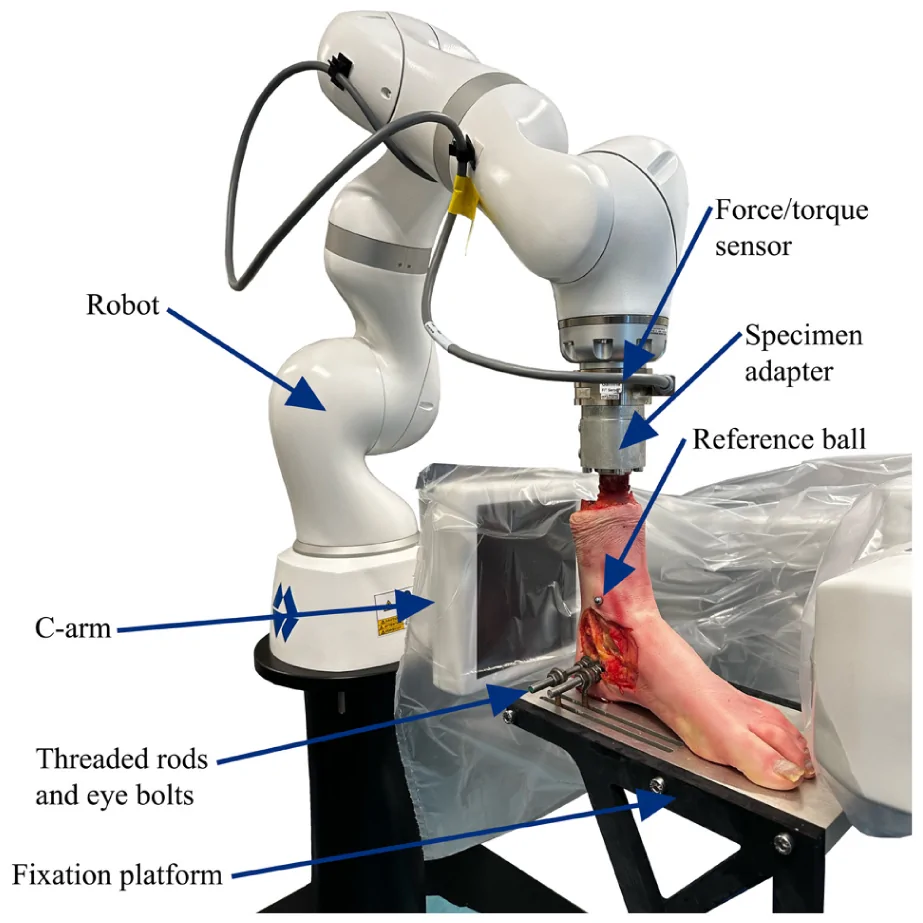
Our experimental setup for robotic ankle stability testing.

We used a floor-mounted serial robot (LBR Med; KUKA, Augsburg, Germany) equipped with a 6-axis Force/Torque Sensor (FTS) (Gamma, ATI Industrial Automation) to perform the biomechanical tests (Figure 2). The FTS measured loads (forces and torques) acting on the specimens, whereas the robot applied loads and measured motion. The FTS has a resolution of 1/20 N and 1/800 Nm, whereas the robot has a pose repeatability of 0.03 mm (ISO 9283). After fixing the calcaneus rigidly to the testing platform, the proximal tibia was fixed to the distal robot arm/force-torque sensor. The specimens were mounted with the ankle joint in neutral position regarding rotation, flexion, and varus/valgus. Fluoroscopy was performed by a mini–C-arm (OEC Elite MiniView; GE Healthcare). An 8-mm metal ball was attached to the medial malleolus for reference size on the radiographs.

### Biomechanical Testing Protocol

The robot applied a constant axial load of 45 N to the tibia throughout all sequences. A modest axial load was selected to limit the load-dependent intrinsic stability caused by the congruency of the ankle joint. Before each test, the robot centralized the ankle joint to achieve 0 N along the anterior-posterior and medial-lateral axes of the ankle, and the neutral position was verified by fluoroscopy for every native specimen. The stability tests were performed with the ankle in neutral position. The force-guided robot performed the biomechanical tests by moving the tibia relative to the fixed calcaneus. Four tests for ankle stability were performed:

1. Lateral translation (talar shift)
2. External rotation
3. Internal rotation
4. Valgus (talar tilt)

**Figure 3.**
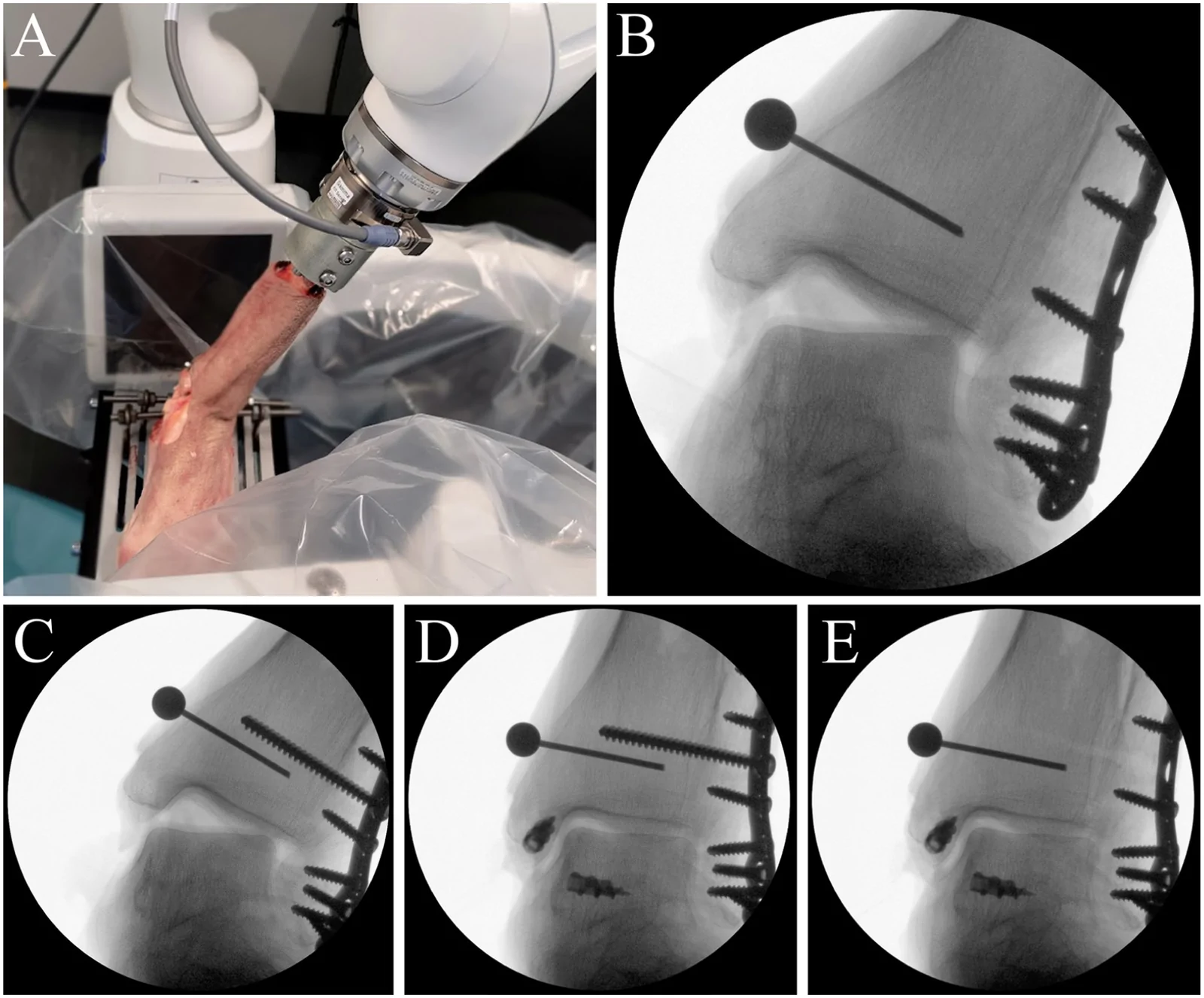
The figure shows a valgus test of an ankle specimen. (A) Overview of the test setup when performing the valgus test. (B) Radiographs from valgus testing of an SER4b injury model with plate fixation. (C) Plate fixation + syndesmotic screw. (D) Plate fixation + syndesmotic screw + deltoid ligament repair. (E) Plate fixation + deltoid ligament repair.

The lateral translation test applied a 30-N force, shifting the tibia medially. Forces arising in the anteroposterior axis were ignored, and movements of the tibia were purely axial or lateral-medial. Rotational and valgus tests were performed by rotating about the respective axes until a test limit of either torque of 2 Nm, 25° of valgus or 40° of external or internal rotation was reached. The industrial robot measured the combined movements of the talocrural and subtalar joints. Isolated movement in the talocrural joint in lateral translation and valgus tests was measured on radiographs recorded at test limits as talar shift and talar tilt, respectively. A valgus test is demonstrated in Figure 3. All specimens were tested sequentially in the following order, as native and 4 different injury model repair states:

1. Native ankle specimen
2. SER4b injury model with fibular plate fixation
3. SER4b injury model with combined plate fixation and tricortical transsyndesmotic screw fixation
4. SER4b injury model with combined plate fixation, syndesmotic screw fixation, and deltoid ligament repair
5. SER4b injury model with combined plate fixation and deltoid ligament repair

### Specimen Injury Models and Surgical Procedures

The specimens were secured to the platform and robot, with the ankle in neutral position, during all repairs and injury procedures. We kept the specimens attached between subsequent tests and interventions to guarantee consistent placement. The fracture model was made by a power saw with an oblique cut from posterolateral proximally ending through the medial anterior fibular cortex at the talocrural joint line. The deltoid ligament was fully transected according to Jeong et al’s description of the most common injury pattern,^18^ with the deep posterior tibiotalar ligament torn close to its talar insertion, and the anterior part off the anterior colliculus of the malleolus medialis (Figure 1). This injury pattern matches our clinical experience. Preliminary results from an ongoing RCT on treatment of deltoid ligament injuries in SER4b injuries report that 22 of 25 patients who underwent repair of the deltoid ligament had complete tears of posterior structures at their talar insertion, and only 2 of 25 were torn from their malleolar attachment.^19^

**Figure 4.**
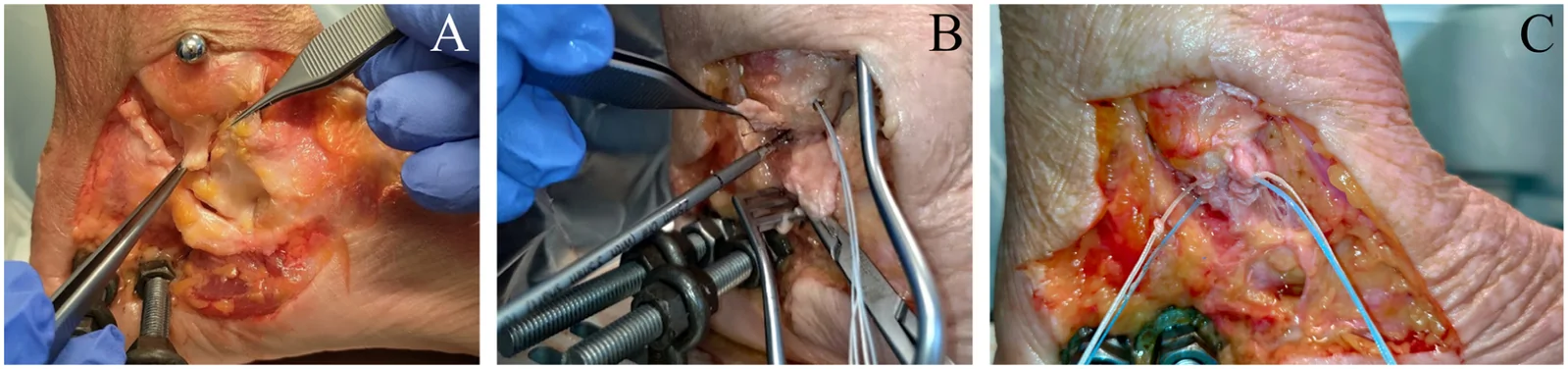
The figure shows the deltoid ligament repair technique. (A) The anterior part of the deltoid ligament was re-attached to the colliculus anterior of the medial malleolus, and the posterior part of the deltoid ligament was re-attached to the medial side of the talus, just inferior to the colliculus posterior of the medial malleolus. (B) A double-threaded suture anchor was screwed into each insertion site, and the different parts of the deltoid ligament were secured with both sutures to their respective anchor and insertion sites (C).

In line with Lauge-Hansens report,^1-3^ the anterior and posterior inferior tibiofibular ligaments were cut completely, and the interosseous ligament was divided just above the joint line. The Weber B fracture model was reduced and fixed with an anatomic implant with angular stability (EVOS 3.5 mm; Smith & Nephew). Syndesmotic reduction was performed openly with visual control of the anterior tibiofibular congruence.^20^ During manual compression, as described by Park et al,^21,22^ we put a 3.5 ×40-mm tricortical transsyndesmotic screw 1 to 2 cm above the ankle joint line. The deltoid ligament was repaired by suturing the deep posterior tibiotalar ligament back to its insertion in the talus using a double-threaded 4.5-mm corkscrew anchor (Arthrex), placed just inferior to the posterior colliculus of the medial malleolus. The deep posterior deltoid ligament is the strongest one of the 3 constant parts of the deltoid ligament complex.^23-25^ The anterior deltoid ligament parts were sutured to a second anchor in the anterior colliculus of the medial malleolus (Figure 4). When tightening the knots of the deltoid ligament sutures, we used a knot-pusher to get a satisfactorily firm repair. Eventually, the trans-syndesmotic screw was fully extracted before the final test.

### Outcomes

The robot measured the total movement between the tibia and the calcaneus, including both the talocrural and subtalar joints. Radiographs were used to measure isolated talocrural joint movement. The primary outcome was defined as the mean difference in talar shift (recorded in millimetres) comparing the Weber B/SER4b injury model with its different repair combinations to the native ankle. Talar shift was estimated as absolute medial clear space (MCS) measurements recorded in millimetres on ankle mortise view radiographs. Consistent with previous descriptions, we defined the MCS as the distance between the medial border of the talus and the lateral border of the medial malleolus on a line parallel to and 5.0 mm below the talar dome.^7,26,27^ The minimal clinically important difference (MCID) for defining talar shift instability was defined as 1.0 mm.^28,29^ Secondary outcomes included radiographic talar valgus tilt (degrees) in the talocrural joint and robotic measurements of lateral translation (millimetres), valgus (degrees), and internal and external rotation (degrees) in the ankle and subtalar joints. Radiographic talar tilt was measured as the angle between the tibial and the talar articular surfaces in the talocrural joint on calibrated fluoroscopic recordings.^17^ The first author interpreted all radiographs. Blinding was not possible because of the use of metal suture anchors. Sectra IDS7, version 26.2 (Sectra AB), was used for radiographic analysis.

### Statistical Analysis

Descriptive statistics were presented using means and corresponding SDs. To test pairwise differences in means across groups, we estimated linear mixed models with random intercepts for study specimens to account for dependency in the data. The a priori power calculation assumed an MCID in means between groups of 1 mm in the talar shift test, with an SD of 0.6 mm.^6,7,11,17^ To achieve a power of 80% and a significance level of 5%, the number of specimens needed was estimated to 8. To account for the possibility of ankle specimens with previously unknown exclusion criteria, such as injuries with sequelae or osteoarthritis, a total of 11 ankles were tested in the study. Stata 18.0 (StataCorp) was used for statistical analysis.

## Results

### Specimens

Three ankle specimens were excluded because of significant ankle osteoarthritis (n = 1), arthrofibrosis (n = 1) and fracture in the medial malleolus during robotic testing caused by a misplaced screw anchor (n = 1). Eight ankle specimens were included in the final analyses. The average age at death was 71 years (range 55-87 years), 2 were female and 6 male, and all 8 were left ankles.

### Biomechanical Tests

**Figure 5.**
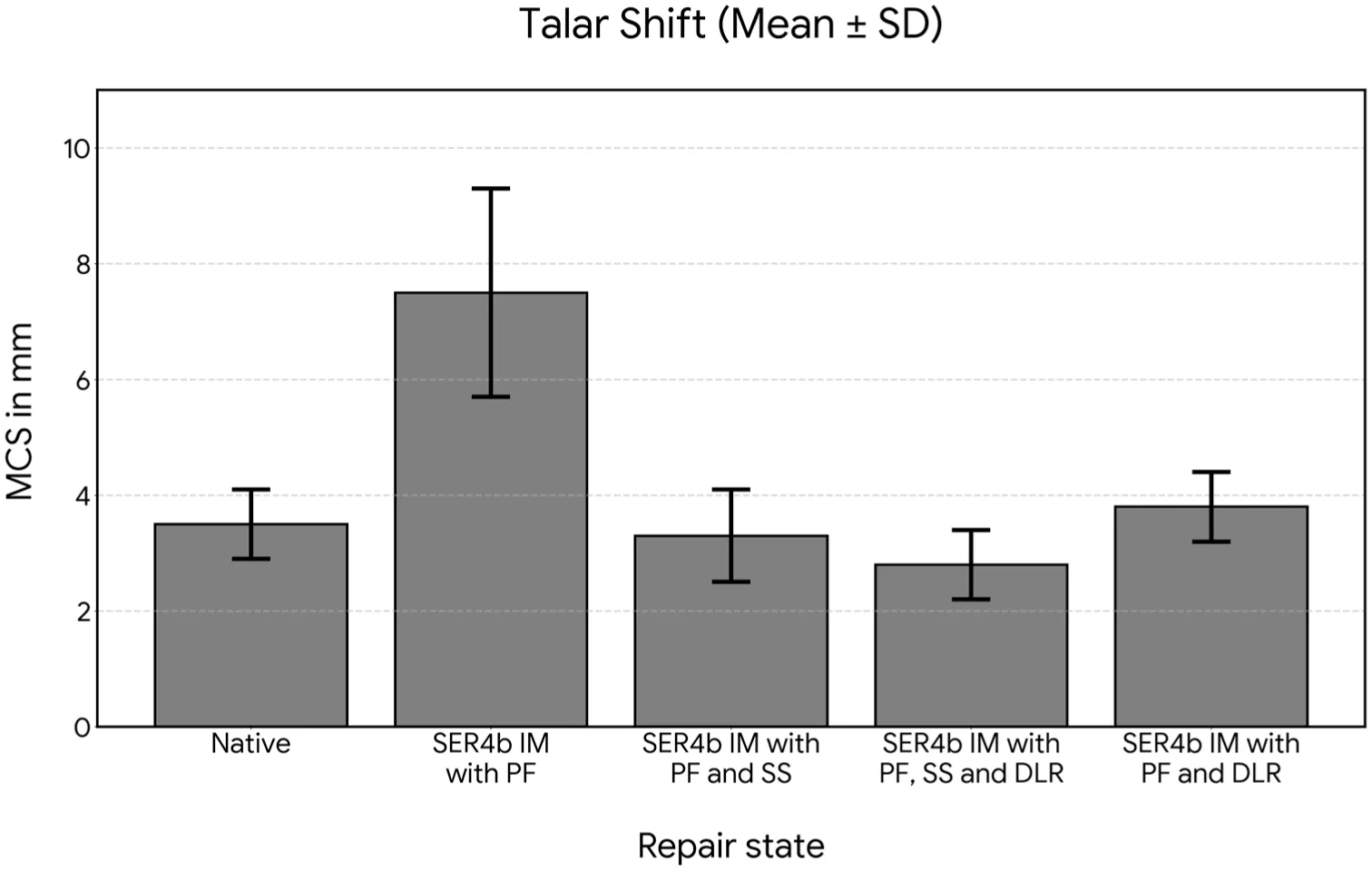
Talar shift, bar plot of data from Table 1. Fluoroscopic medial clear space (MCS) absolute measurements in millimetres, under 45 N axial load and lateral translation stress of 30 N. Error bars reflecting average values ± 1 SD. Graph made using Python 3.13 (python.org). DLR, deltoid ligament repair; IM, injury model; PF, plate fixation; SS, syndesmotic screw.

Test results of native ankles showed a mean talar shift of 3.5 mm (SD 0.6 mm). Lateral translation stability was achieved with both combined plate fixation and syndesmotic screw (mean talar shift of 3.3 mm [SD 0.8]), combined plate fixation and deltoid ligament repair (mean talar shift of 3.8 mm [SD 0.6 mm]), and combined plate fixation, syndesmotic screw, and deltoid ligament repair (mean talar shift of 2.8 mm [SD 0.6 mm]) (Table 1, Figure 5). All surgical methods, except plate fixation only, achieved a difference in talar shift within the pre-defined MCID of 1.0 mm, when compared to native ankles (Table 2).

**Table 1.** Stability Measurements in 8 Ankles Tested Sequentially in Neutral Position as Native, SER4b Injury Models (SER4b IM) With Plate Fixation (PF), SER4b IM With Combined PF and Syndesmotic Screw (SS), SER4b IM With Combined PF, SS, and Deltoid Ligament Repair (DLR) and SER4b IM With Combined PF and DLR.

Test	Native, Mean (SD)	SER4b IM With PF, Mean (SD)	SER4b IM With PF and SS, Mean (SD)	SER4b IM With PF, SS and DLR, Mean (SD)	SER4b IM With PF and DLR, Mean (SD)
Talar shift^a^, mm	3.5 (0.6)	7.5 (1.8)	3.3 (0.8)	2.8 (0.6)	3.8 (0.6)
Lateral translation^b^, mm	4.2 (0.8)	8.7 (2.0)	4.7 (1.3)	4.0 (0.9)	5.2 (0.8)
Talar valgus tilt^a^, degrees	2.9 (1.5)	21.7 (4.5)	21.0 (4.2)	5.9 (2.3)	5.6 (2.7)
Valgus^b^, degrees	5.6 (1.3)	24.1 (1.7)	24.1 (1.9)	10.4 (4.5)	8.6 (2.8)
Internal rotation^b^, degrees	9.8 (1.8)	29.9 (5.5)	29.1 (5.4)	18.8 (4.8)	18.8 (2.2)
External rotation^b^, degrees	10.9 (2.8)	28.3 (4.1)	26.8 (3.4)	12.2 (5.4)	16.1 (4.4)

Abbreviation: SER, supination external rotation.

a Radiographic measurement.

b Robot measurement.

**Table 2.** Stability Differences in 8 Ankles Tested Sequentially in Neutral Position Comparing SER4b Injury Models (SER4b IM) With Plate Fixation (PF) to Native, SER4b IM With Combined PF and Syndesmotic Screw (SS) to Native, SER4b IM With Combined PF, SS, and Deltoid Ligament Repair (DLR) to Native and SER4b IM With Combined PF and DLR to Native.

	SER4b IM With PF Compared to Native			SER4b IM With PF and SS Compared to Native		
Test	MD	(95% CI)	*P*	MD	(95% CI)	*P*
Talar shift^a^, mm	4.1	(3.4 to 4.7)	<.001	−0.2	(−0.8 to 0.5)	.573
Lateral translation^b^, mm	4.5	(3.8 to 5.2)	<.001	0.6	(−0.1 to 1.3)	.115
Talar valgus tilt^a^, degrees	18.8	(16.9 to 20.8)	<.001	18.1	(16.1 to 20.0)	<.001
Valgus^b^, degrees	18.5	(16.1 to 21.0)	<.001	18.5	(16.0 to 20.9)	<.001
Internal rotation^b^, degrees	20.1	(16.6 to 23.5)	<.001	19.3	(15.8 to 22.8)	<.001
External rotation^b^, degrees	17.4	(14.0 to 20.7)	<.001	15.9	(12.6 to 19.2)	<.001
	SER4b IM With PF, SS, and DLR compared to Native			SER4b IM With PF and DLR Compared to Native		
	MD	(95% CI)	*P*	MD	(95% CI)	*P*
Talar shift^a^, mm	−0.6	(−1.3 to 0.01)	.055	0.30	(−0.4 to 1,0)	.367
Lateral translation^b^, mm	−0.2	(−0.9 to 0.5)	.657	0.97	(0.3 to 1.7)	.007
Talar valgus tilt^a^, degrees	3.0	(1.0 to 4.9)	.003	2.74	(0.8 to 4.7)	.006
Valgus^b^, degrees	4.8	(2.4 to 7.3)	<.001	3.06	(0.6 to 5.5)	.014
Internal rotation^b^, degrees	9.0	(5.5 to 12.5)	<.001	8.96	(5.5 to 12.4)	<.001
External rotation^b^, degrees	1.3	(−2.0 to 4.7)	.427	5.20	(1.9 to 8.5)	.002

Abbreviations: MD, mean difference; SER, supination external rotation.

a Radiographic measurement.

b Robot measurement.

**Figure 6.**
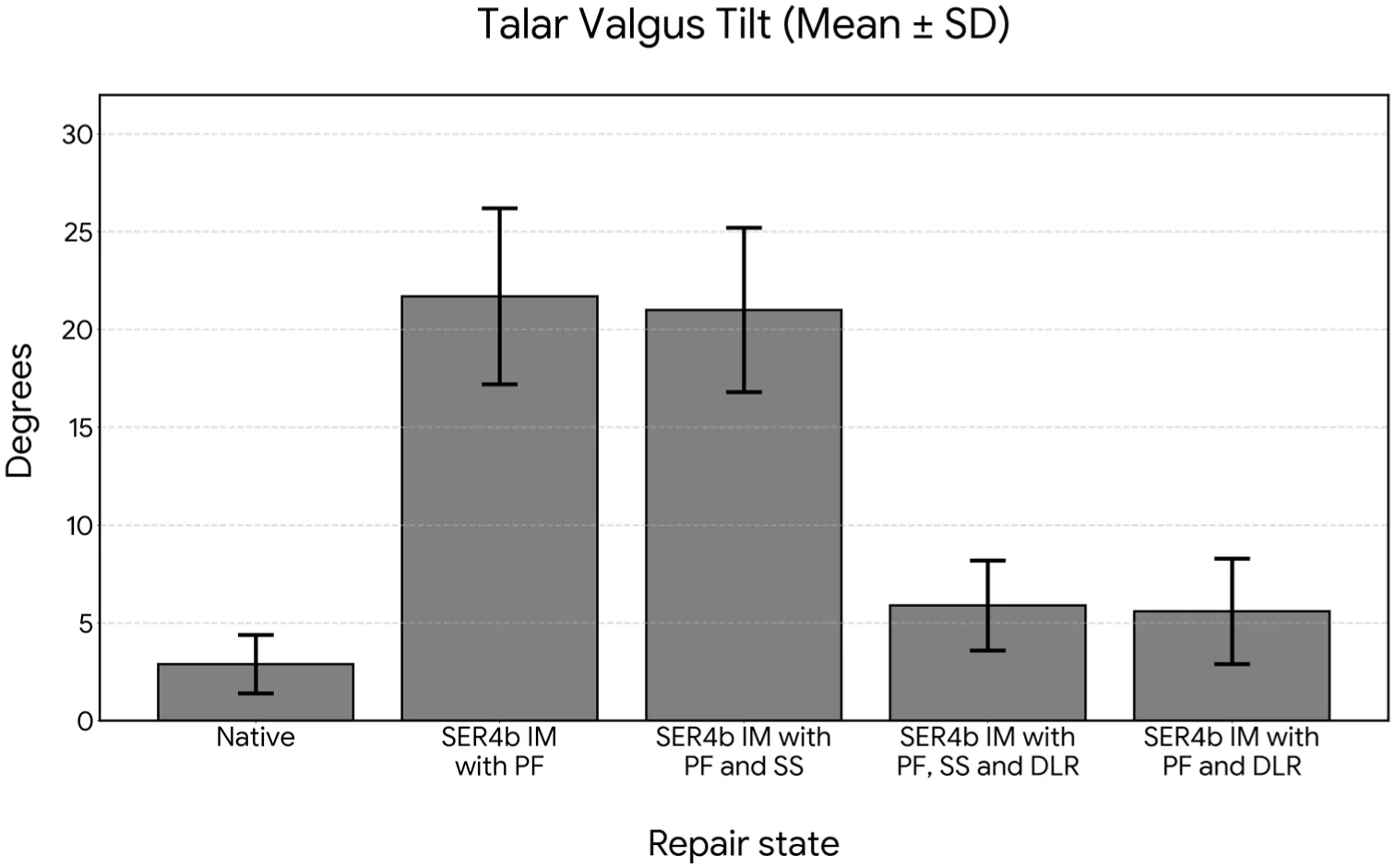
Talar valgus tilt, bar plot of data from Table 1. Tibiotalar angle measurements from fluoroscopic outprints in degrees, recorded when what was first reached of a robotic valgus torque of 2 Nm or 25° of valgus measured by the robot under a constant 45 N axial load. Error bars reflecting average values ± 1 SD. Graph made using Python 3.13 (python.org). DLR, deltoid ligament repair; IM, injury model; PF, plate fixation; SS, syndesmotic screw.

**Figure 7.**
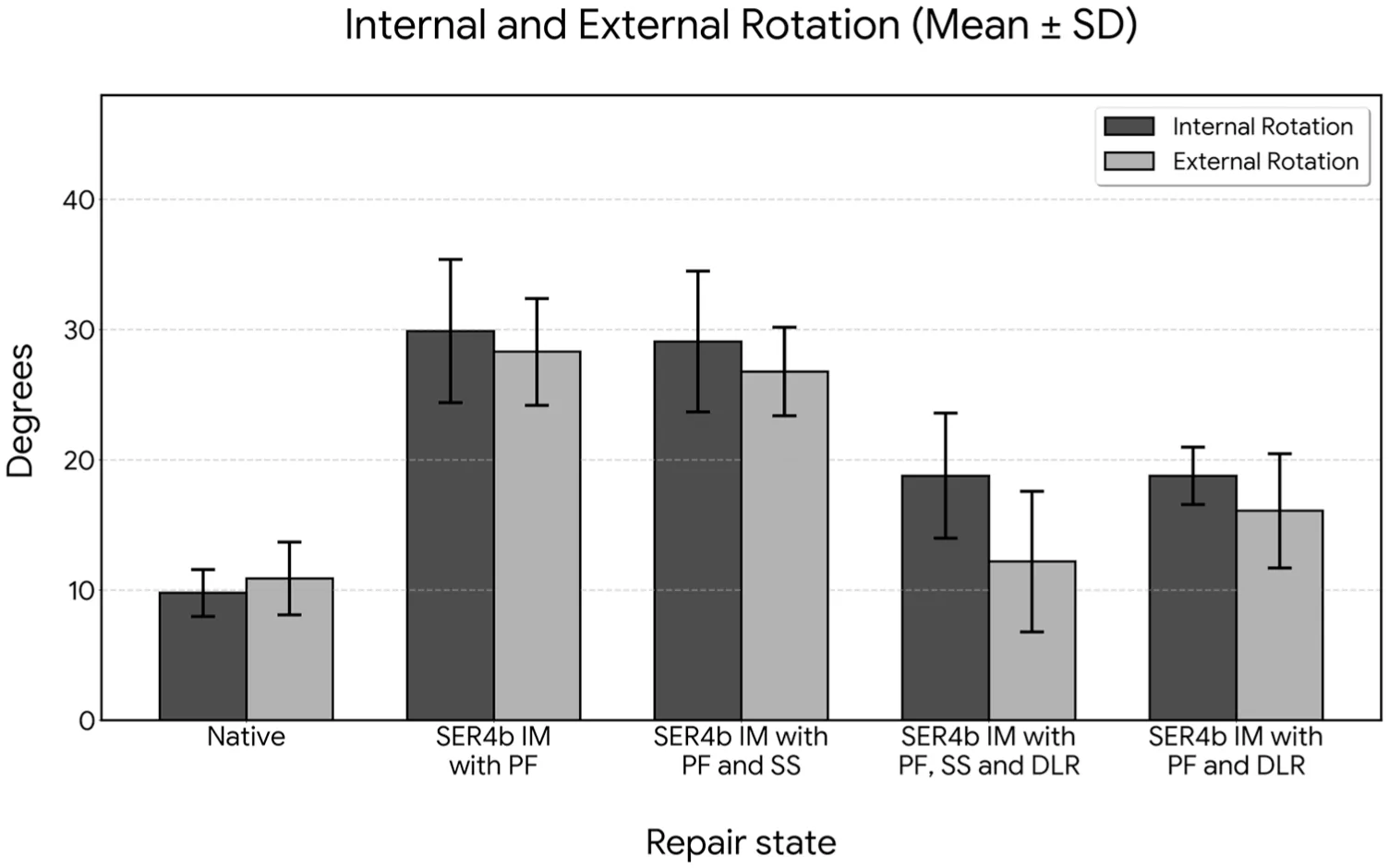
Rotational stability, bar plots of data from Table 1. Internal rotation dark and external rotation light grey bars. Robotic rotational measurements in degrees, under 45 N axial load and what was first reached of a robotic rotational torque of 2 Nm or 40° of internal or external rotation. Error bars reflecting average values ± 1 SD. Graph made using Python 3.13 (python.org). DLR, deltoid ligament repair; IM, injury model; PF, plate fixation; SS, syndesmotic screw.

For the secondary outcomes, combined plate fixation and deltoid ligament repair demonstrated superior stability to combined plate fixation and syndesmotic screw in both valgus and rotational stability testing (Table 3, Figures 6 and 7). The mean difference in talar valgus tilt was 15.3° (95% CI 13.2-17.5, *P* < .001), internal rotation 10.3° (95% CI 6.7-15.0, *P* < .001) and external rotation 10.7° (95% CI 7.1 to 14.3, *P* < .001), all in favour of combined plate fixation and deltoid ligament repair.

**Table 3.** Stability Differences in 8 Ankles Tested Sequentially in Neutral Position Comparing SER4b Injury Models (SER4b IM) With Combined Plate Fixation (PF) and Syndesmotic Screw (SS) to SER4b IM With Combined PF and Deltoid Ligament Repair (DLR).

	SER4b IM With PF and SS Compared to SER4b IM With PF and DLR		
Test	MD	(95% CI)	*P*
Talar shift^a^, mm	−0.5	(−0.8 to −0.2)	.002
Lateral translation^b^, mm	−0.4	(−0.7 to −0.1)	.010
Talar valgus tilt^a^, degrees	15.3	(13.2 to 17.5)	<.001
Valgus^b^, degrees	15.4	(12.4 to 18.4)	<.001
Internal rotation^b^, degrees	10.3	(6.7 to 14.0)	<.001
External rotation^b^, degrees	10.7	(7.1 to 14.3)	<.001

Abbreviations: MD, mean difference; SER, supination external rotation.

a Radiographic measurement.

b Robot measurement.

Both SER4b injury models with combined plate fixation and deltoid ligament repair and SER4b injury models with combined plate fixation, syndesmotic screw, and deltoid ligament repair demonstrated good stability in all tested directions when compared to native ankles (Table 2).

## Discussion

The key finding of this study is that the repair of a cadaveric SER4b injury model with an anatomic fibular plate and tricortical trans-syndesmotic screw fixation restores lateral translational stability but does not restore rotational or valgus stability. In contrast, deltoid ligament repair combined with plate fixation, provides substantial improvement in overall ankle stability, approximating native ankle mechanics. Interestingly, the combination of all 3 techniques added a modest effect beyond what was achieved by combined deltoid ligament repair and plate fixation alone.

In accordance with previous biomechanical studies, the SER4b injury model with fibular plate fixation only was unstable in all tested directions.^11,12^ When adding a syndesmotic screw, we found a markedly reduced talar shift, within the predefined MCID of 1.0 mm. Similar to the findings of LaMothe et al,^22^ we saw that despite improving lateral translational stability, syndesmotic fixation did not meaningfully reduce rotational or valgus instability. Because the primary outcome, talar shift, was restored within the pre-defined MCID by both fixation strategies, the two approaches were equivalent on the pre-specified measure; the clinically meaningful divergence between them emerges from the secondary outcomes of valgus and rotational stability.

When detected, lateral translation instability is likely of clinical importance. Ramsey and Hamilton’s cadaveric study linked lateral translation of the talus to decreasing talocrural contact area, increased joint contact pressures and the risk of posttraumatic arthritis.^2,28,30,31^ There is limited information on MCID regarding valgus and rotational stability in previous biomechanical studies.^32^ McCormack et al^13^ proposed 7° as the MCID for valgus. Although established reference values are lacking, the increases of 18° valgus and 15° to 20° rotational movements observed in our comparisons may represent clinically meaningful changes. We believe that valgus and rotational instability also may be important. Subtle rotational posttraumatic instability is likely more prevalent than obvious lateral translation instability. Still, we miss validated ways of detecting rotational instability in everyday clinical practice. Several studies have investigated rotational instability without defining limits for what is clinically important.^12,33,34^ Hunt et al^35^ showed that pathologic rotational movements produce abrupt increases in joint contact pressure, reduced size and relocation of centre of tibiotalar contact area in ankle injury models, and proposed that this could lead to post-traumatic arthritis.

Similarly to only few others, Dalen et al^11-13^ reported a substantial stabilizing effect of combined fibular plate fixation and repair of the deltoid ligament injury in an SER4b injury model. Butler et al^12^ made a more anterior insertion of the deltoid ligament structures, whereas Dalen et al^11^ focused on the deep posterior tibiotalar ligament (dPTTL). Their findings are comparable with our present results, in spite of differences in the ligament repair techniques used. In our present study, both anterior and posterior components of the deltoid ligament were repaired, which represents our standard clinical practice. The combination of fibular plate fixation and deltoid ligament repair resulted in substantial improvement in valgus stability and both internal and external rotational stability, in addition to lateral translation stability. Our findings suggest that deltoid ligament competence is essential for restoring native ankle mechanics after SER4b injuries. When all 3 surgical techniques were combined, ankle stability closely approximated that of the native ankle. Lateral translation stability was already restored within pre-defined limits after lateral plating and deltoid ligament repair. The addition of a trans-syndesmotic screw improved resistance to talar shifting forces even further but did not affect valgus stability and had only a minor effect on external rotation.

This study is the first to biomechanically evaluate 3 different combinations of surgical repair for the SER4b injury and incorporate a broader range of stability tests and by both radiographic and robotic measurements. As such, our study provides a more rigorous evaluation and producing novel evidence on multiplanar instability on SER4b injury models. The importance of the ligamentous contributions to joint stability may be overestimated in our test setup with lower stabilizing axial load and relatively higher dislocating forces. Rotational stability may increase by a load-dependent joint congruency effect. We appreciate the need for similar fracture models and repair states to be tested under physiological loading. Though, we know from weightbearing radiographic recordings in clinical practice, using the patient’s half body weight on the injured limb, that the ankle joint with an SER4b injury loses position during a purely axial loading.

True to the Lauge-Hansen classification, our SER4b injury model involved complete sectioning of the anterior and posterior inferior tibiofibular ligament, interosseous ligament, and the deep deltoid ligament, representing a particularly unstable configuration.^3^ Most likely, this partly reflects in vivo clinical Weber B/SER4b ankle fractures identified by weightbearing radiographs. We expect some degree of variety in the severity of syndesmotic ligament injury.^11,12,18,36^ MRI studies investigating this may give further insight. Our clinical experience, including sequential perioperative stability testing, is that some anterior syndesmotic ligament injuries are only partial. Maybe the posterior inferior tibiofibular ligament in some cases may be intact. If so, our fracture model may be more unstable than the average SER4b injury. Then, our fracture model and test setup could overestimate the need for and impact of syndesmotic and deltoid ligament repair.

### Clinical Relevance

The results from this study suggest that syndesmotic fixation can substitute deltoid ligament repair only in restoring lateral translation stability. Deltoid ligament repair gives a more general impact on joint stability in Weber B/SER4b injuries.

### Limitations

This study has several limitations. Generally, although cadaveric studies enhance our understanding of ankle injury biomechanics, their findings should be applied to clinical practice with caution. Our study is exploratory. Current knowledge about MCIDs is limited for valgus and rotational instability. We did not predefine thresholds or make power calculations for our secondary endpoints, rotational and valgus instability. The injury model is strictly controlled and therefore may not fully reflect the complexity and variability of injuries observed in living patients.^11,12,18,36^ Strictly, our findings are only valid under the loading conditions and dislocating forces applied in our study.

As with all cadaveric studies, the absence of biological healing and effect of natural loading during postoperative rehabilitation limits the direct translation of these results to clinical settings. Specifically for this study, robotic testing measured motion in both the ankle and subtalar joints. Radiographic measurements were isolated to the tibiotalar joint. Testing was performed only in the neutral ankle position, and cyclic loading was not evaluated. Anteroposterior translation tests were left out, mainly because we do not usually apply them in our clinical practice, except for stability assessment in cases of posterior malleolar fragments or lateral ligament injuries.

The ligament repairs in this study were likely technically easier to perform than in clinical practice. When repairing in vivo naturally fringed deltoid ligament injuries, the suture purchase may be more difficult than when they are schematically cut in the lab, even if most patients selected for this procedure will be younger than our cadaveric specimens. As our different repair constructs were tested in the same order for all specimens, the potential effect from this could be that the plate and screw fixation, suture, and the specimen may be weakened by repeated test cycles before the last test rounds. The 2 repair states tested at the end were prone to be affected by this but came out as the most stable. We saw no sign of implant loosening during the tests performed. Beyond this, we did not control for cumulative soft tissue fatigue or microstructural damage.

In spite of being a simpler technical procedure, trans-syndesmotic fixations by buttons or screws are not infallible because of a considerable risk of syndesmotic malreduction.^37^ Leaning on the assumption that the advantages of the syndesmotic button is more relevant for the outcome after patient rehabilitation than immediate postoperative stability measurements, we used a tricortical screw.^38,39^ The challenges of syndesmotic repair and reduction is not a topic of this article.

## Conclusion

In this cadaveric Weber B/SER4b fracture model with complete deltoid ligament sectioning, both syndesmotic screw fixation and deltoid ligament repair restore lateral translational stability when combined with fibular plate fixation, meeting the pre-defined minimal clinically important difference. In contrast, only deltoid ligament repair—in which both the deep posterior tibiotalar and anterior deltoid components were addressed—substantially restored rotational and valgus stability. These findings suggest that syndesmotic screw fixation may not be a sufficient substitute for this repair construct when multiplanar stability is the surgical goal, and support further clinical investigation of deltoid ligament repair in this injury pattern.

## Supplemental Material

sj-pdf-1-fai-10.1177_10711007261445948 – Supplemental material for Multiplanar Stability After Syndesmotic Screw Fixation vs Deltoid Ligament Repair in a Cadaveric Weber B/SER4b Ankle Fracture Model With Fibular Plate Fixation Supplemental material, sj-pdf-1-fai-10.1177_10711007261445948 for Multiplanar Stability After Syndesmotic Screw Fixation vs Deltoid Ligament Repair in a Cadaveric Weber B/SER4b Ankle Fracture Model With Fibular Plate Fixation by Esten K. Ø. Haanæs, Andreas F. Dalen, Martin G. Gregersen, Aleksander Skrede and Marius Molund in Foot & Ankle International
